# Not all enhancing lesions are tumor recurrence: Foreign body granulomas after glioma resection

**DOI:** 10.1016/j.bas.2026.106140

**Published:** 2026-06-23

**Authors:** Maximilian Scheer, Julian Prell, Christian Mawrin, Sandra Leisz, Stefan Schob, Stefan Rampp, Sebastian Simmermacher

**Affiliations:** a Department of Neurosurgery, University Hospital Halle, Germany; b Department of Neuropathology, University Magdeburg, Germany; c Department of Neuroradiology, Department for Neuroradiology, Klinikum St. Georg, Leipzig, Germany; d Department of Neurosurgery, Institute of Neuroradiology, University Hospital Erlangen, Germany; e Department of Neurosurgery, University Hospital Heidelberg, Germany

**Keywords:** Textiloma, Foreign body granuloma, Gossypiboma, Neurooncology, Glioma, Recurrence

## Abstract

**Introduction:**

Postoperative imaging surveillance is essential in the management of glioma patients. However, new or progressive contrast-enhancing lesions do not always indicate true tumor recurrence. Foreign body granulomas (FBG), also known as textilomas, may arise as inflammatory reactions to hemostatic or sealing materials used during surgery and can closely mimic tumor progression on MRI. Distinguishing between these entities remains a major diagnostic challenge with important therapeutic implications.

**Methods:**

We retrospectively analyzed glioma re-operations performed for suspected recurrence at a tertiary neurosurgical center between 2016 and 2020. Demographic data, tumor histology, intraoperative foreign material, adjuvant therapy, and time to re-operation were assessed. Histological findings were classified as recurrence or no tumor. Preoperative MRI was reviewed according to RANO criteria, with emphasis on diffusion-weighted imaging and apparent diffusion coefficient (ADC) values. Univariate analyses were conducted.

**Results:**

Of 211 glioma patients, 38 (18 %) underwent re-operation for suspected recurrence. Histological recurrence was confirmed in 27 cases (71.1 %), while 11 (28.9 %) showed no tumor. Foreign material was identified in three non-tumor cases; the remaining showed post-inflammatory or unclear changes. All non-tumor cases had been radiologically classified as progressive disease. ADC values tended to be lower in the non-tumor group, suggesting diffusion restriction, but without statistical significance. Oxidized cellulose was frequently used in both groups**.**

**Conclusion:**

FBG are a relevant and likely underrecognized differential diagnosis in suspected glioma recurrence. Conventional MRI and RANO criteria alone may be insufficient. Diffusion-weighted imaging may provide additional clues but is not definitive. Increased awareness and multidisciplinary evaluation are essential to avoid unnecessary interventions and overtreatment.

## Highlights

• Relevant misdiagnosis rate: In 28.9 % of re-operations for suspected progression, no tumor was found, but rather foreign body reactions (FBG) or post-inflammatory changes.
• Limitations of established criteria: All non-tumor cases were preoperatively classified as progressive disease according to RANO criteria, highlighting the insufficiency of conventional MRI assessment alone.
• Imaging with limited discriminatory value: Diffusion-weighted imaging (ADC) tended to show restricted diffusion even in non-tumor lesions, but without statistically significant differentiation.
• Clinical implication: Foreign body granulomas are a relevant and likely underrecognized differential diagnosis, requiring heightened awareness and multidisciplinary interpretation to avoid overtreatment and unnecessary re-operations.

## 1 Introduction

Gliomas are a heterogeneous group of brain tumors. In general, surgical treatment is a mainstay of therapy for these. Each procedure, however, carries the risk of postoperative complications such as wound infection, seizures, or postoperative bleeding (Weller et al., 2023). Careful hemostasis at the end of surgery is essential to ensure that the risk of secondary bleeding is minimized (Dobran et al., 2022; Weller et al., 2023) (see Table 1).

**Table 1:** Baseline features of Patients with Re-Operation.

	Recurrence	No Evidence of Tumor	p value
**Number** (n)	27	11	
**Age** (Mean) [Range]	57.1 [35-76]	52.9 [32-71]	0.310
**Gender**			0.283
Female	8	6	
Male	19	5	
**Histology**			0.932
WHO Grade 2	8	3	
WHO Grade 3	2	1	
WHO Grade 4	17	7	
**Usage of foreign material** (n, %)			
Tabotamp®	20 (74.1 %)	10 (90.9 %)	0.627
TachoSil®	6 (22.2 %)	1 (9.1 %)	0.627
Gelita®	3 (11.1 %)	0 (0 %)	0.625
No foreign material	3 (11.1 %)	0 (0 %)	0.436
**Time to second surgery** (days, median) [Range]	383 [64-2969]	184 [5-2872]	0.633
**Prior Chemotherapy**	18 (66.6 %)	8 (72.7 %)	1.0
**Prior Radiation**	20 (74.1 %)	8 (72.7 %)	1.0
**ADC values (mm^2^/s x 10^.−6^)** [SD]	812.60 [142.2]	673.87 [86.8]	0.52

For this purpose - or to reduce the risk of a CSF fistula after opening the ventricle - foreign material may be placed in the resection cavity. Examples for these foreign materials include: Gelatin sponges (e.g. Gelita®), oxidized cellulose (e.g. Tabotamp®) or mixed coated patches with fibrinogen/thrombin collagen (e.g. TachoSil®) (Roberto Giammalva et al., 2021).

However, the introduction of foreign material always carries the risk of triggering a foreign body reaction (FBR) (Anderson et al., 2008). This FBR leads to the adhesion of monocytes and macrophages, macrophage fusion and the formation of giant cells (Eslami-Kaliji et al., 2023; Lin et al., 2014). After neurosurgical procedures, the consequences of this inflammatory reaction, called foreign body granulomas (FBG) or textilomas, have been observed in almost all types of surgery (Jaramillo-Jiménez et al., 2020; Kothbauer et al., 2001; Ribalta et al., 2004). These FBG are sometimes also referred to as gossypibomas (Picón-Jaimes et al., 2019; Hannachi et al., 2024). Examples where FBG have been observed include hemicraniectomy (duraplasty), procedures such as microvascular decompression, aneurysm wrapping or clipping, ventriculoperitoneal shunt placement, surgery for intracerebral hemorrhage, or after embolization of an arteriovenous malformation (Mohammed et al., 2023; Dinesh et al., 2008; Akhaddar et al., 2018; Lebrun et al., 2014; Hasturk and Basmaci, 2013).

In some cases, FBG have also been described in the literature in neuro-oncology patients (Anderson et al., 2013; Winter et al., 2021; Hannachi et al., 2024). A few case reports indicate that oxidized cellulose was used at the time of the first surgery or that radiochemotherapy was performed prior to the second surgery (Nakayama et al., 1994; Anderson et al., 2013; Forst et al., 2016; Al-Afif et al., 2018). What remains unclear is whether there is a causal relationship between FBG and the used material or radiation or the combination of both, as the time to reoperation in which FBG was detected varies widely, ranging from two weeks to 20 years (Akhaddar et al., 2018). The use of non-absorbable materials has been known to result in certain observations in other disciplines (Itoga et al., 2021; Wang et al., 2025; Yoon et al., 2025).

FBGs are usually detected as part of routine MRI follow-up, which is typically performed every 3-6 months depending on the tumor entity. The Response Assessment in Neuro-Oncology (RANO) criteria are used to assess imaging and tumor status, with 4 stages: complete remission, partial remission, stable disease or progression. The presence of new lesions on imaging and progression on contrast-enhanced T1 and T2/FLAIR sequences are evaluated and the patient's clinical status and use of corticosteroids is taken into account (Ellingson et al., 2024). However, with these criteria, it is sometimes difficult to distinguish a FBG from a tumor recurrence or tumor progression. Despite advanced imaging modalities, including MR spectroscopy, perfusion and dynamic contrast-enhanced MRI, reliable differentiation remains challenging, as foreign body granulomas may exhibit largely benign imaging characteristics while even [^18^F]fluorocholine PET/CT can yield false-positive findings that mimic tumor progression. (Garciarena et al., 2013; Jang et al., 2010; Rudolphi-Solero et al., 2023).

**Fig. 1:**
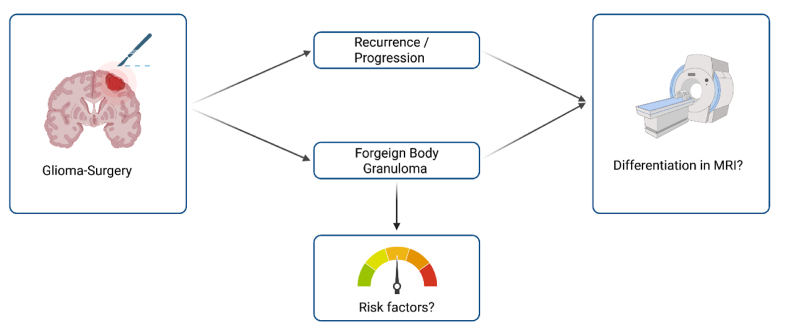
Schematic representation of the underlying rationale of this study.

In this study, we reviewed all glioma recurrence surgeries over a four-year period for the use of foreign material in the initial surgery, demographics, potential concomitant factors such as radiation or chemotherapy, and the occurrence of FBG. A schematic representation of the basic idea is shown in Fig. 1.

## 2 Methods

A retrospective analysis of all surgeries for suspected recurrence in glioma patients performed in our department between 2016 and 2020 was performed. Demographic data such as age or gender were recorded, as well as the tumor entity of the first surgery. In addition, the interval to the second surgery was recorded and, if applicable, the foreign material implanted during the first surgery. The follow-up treatment of the tumors, i.e. whether radiotherapy or chemotherapy was carried out, was recorded, as was the interval until reoperation. The histological work-up was performed according to the WHO classification of tumors of the central nervous system valid at the time of surgery (Louis et al., 2016). The histological results were categorized into two groups: the detection or non-detection of tumor tissue. The second category was further subdivided into the following: detection of foreign material; post-inflammatory changes; and unclear histology.

Follow-up visits involving clinical assessments and imaging were scheduled according to the diagnosis and ongoing treatment. For WHO Grade 2 tumors, an MRI scan was usually performed after six months. For WHO Grade 3 and 4 tumors, follow-up MRI scans were performed every three months. The MRI images were reviewed by the interdisciplinary tumor board based on the current RANO criteria. For high-grade gliomas, the review looked for an increase in contrast uptake of >25 % along with concurrent T2/FLAIR changes. For low-grade gliomas, an increase of >25 % in the T2/FLAIR sequence was considered progression (Wen et al., 2010; van den Bent et al., 2011). The diffusion sequences, which means the value of the Apparent Diffusion Coefficient (ADC), were also analyzed in this study.

Follow-up examinations were brought forward in cases of clinical deterioration. Repeat surgery was performed on an interdisciplinary basis, taking into account the location of the lesion, the likelihood of complete resection, the patient's general condition and their wishes.

### 2.1 Statistical analysis

For comparison to the two groups, univariate statistics were employed. Normal distribution was evaluated using the Shapiro-Wilk-test. The two-sample *t*-test assuming unequal variances was then used for continuous, normally distributed variables, respectively a Mann-Whitney-U-test for continuous variables with non-normal distribution. Frequencies were evaluated with the fisher's exact test. Correction for multiple comparisons was not used due to the exploratory character of the study.

## 3 Results

**Fig. 2:**
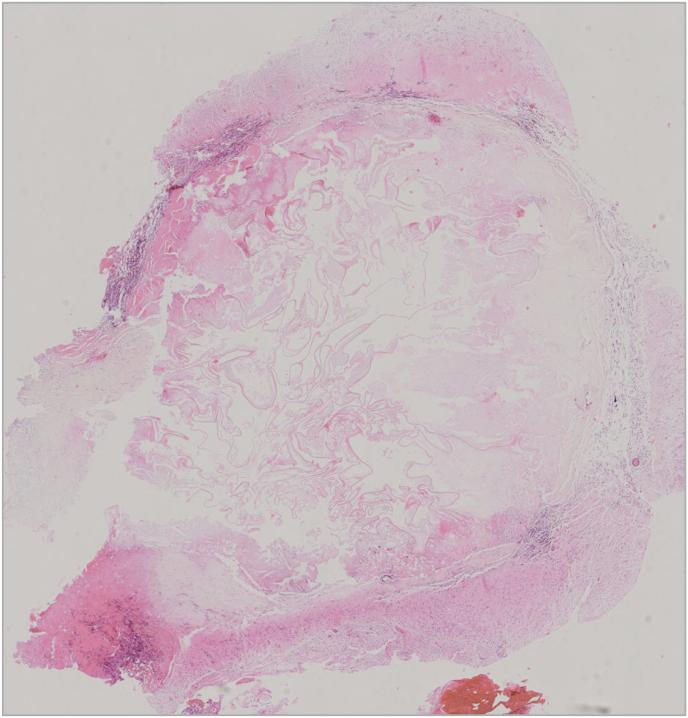
Microscopic image of hematoxylin and eosin staining: Portions of plastic with extensive reactive changes of the surrounding tissue. The microscope image was obtained at 100x magnification.

A total of 211 patients with glioma were treated surgically in our department between 2016 and 2020, of whom 38 (18 %) underwent re-operation for suspected recurrence. Among these re-operations, there were 27 (71.1 %) cases with histological evidence of recurrence (group 1) and 11 (28.9 %) cases in which no tumor material could be detected by conventional microscopy (group 2). Fig. 2 illustrates a typical microscopic finding of a FBG.

**Fig. 3:**
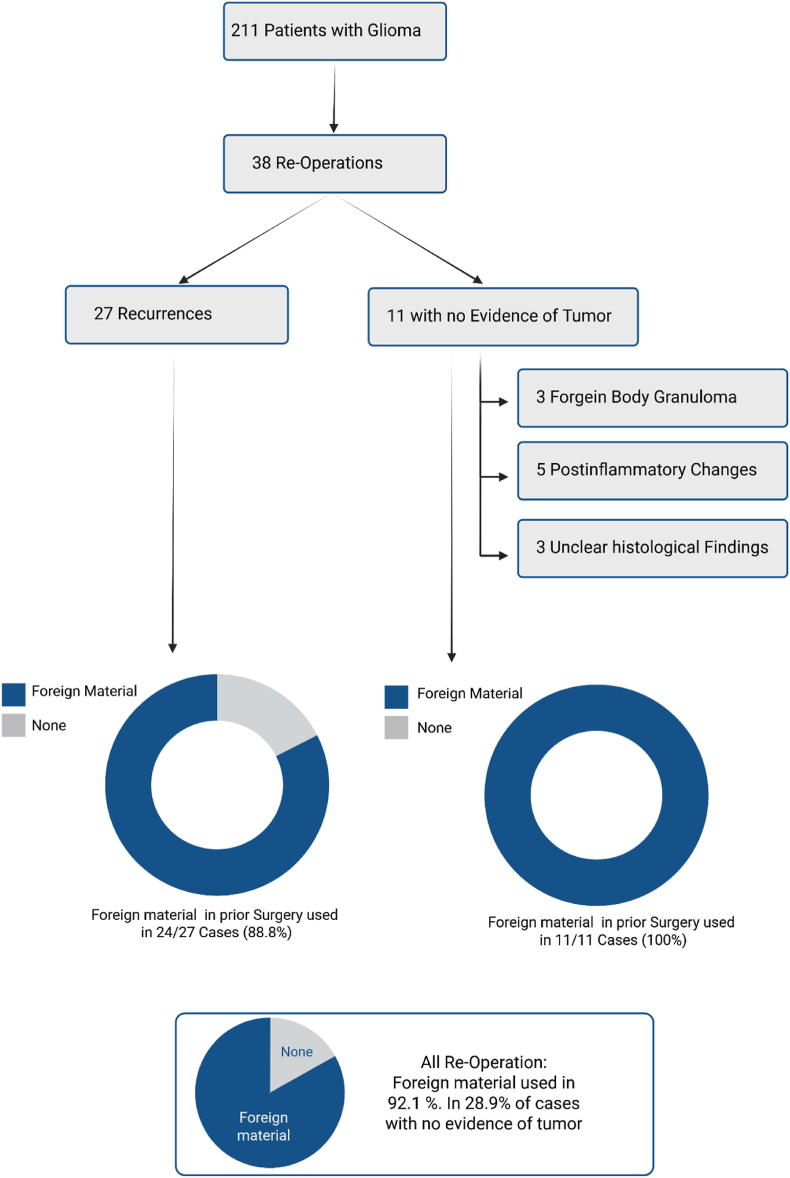
Flow chart of patients with Re-operation.

Foreign material was detected on histological examination in three cases in the group without evidence of tumor recurrence. In five cases post-inflammatory changes were present and in three cases the histological findings were unclear (Fig. 2). The mean age in the group with tumor detection was 57.1 [range: 35-76] years, and in the group without evidence of tumor was 52.9 [32-71] years (difference no significant, p = 0.31, *t*-test) A flow chart of all patients receiving re-operation is shown in Fig. 3.

There were more men in the first group (19/27; 70.3 %). In the second group, the ratio was relatively balanced with 6 female (54.5 %) and 5 male patients (45.5 %), the difference however did not reach statistical significance (p = 0.27, fisher's exact test). The largest proportion of tumors in both groups were WHO grade 4 tumors with 17 tumors (62.9 %) in group 1 and 7 tumors (63.6 %) in group 2. WHO grade 2 tumors were the second most common with 8 tumors (29.6 %) in group 1 and 3 tumors (27.3 %) in group 2. WHO grade 3 tumors were less common with 2 tumors in group 1 and 1 patient in group 2 (distribution differences not significant, p = 1.0, fisher's exact test).

Foreign material was used in 24 (88.8 %) cases in group 1 (6 cases with two different foreign materials). In the group without tumor detection, this was the case in all 11 cases (p = 0.54, fisher's exact test). In both groups, oxidized cellulose (Tabotamp®) was used most frequently. In exact numbers: 20 times (74.1 %) in group 1 and 10 times (90.1 %) in group 2. The time to second surgery was longer in the group with tumor detection with a median of 383 [64-2969] days compared to 184 [5-2872] days in the group without tumor detection, however the difference did not reach statistical significance (p = 0.33, Mann-Whitney-U-test). The data for PFS, histology over time and OS are presented in Tables S1 and S2.

The majority of patients received adjuvant therapy. This was the case for 20 patients (74.1 %) in group 1 and 8 patients (72.7 %) in group 2 (p = 1.0, fisher's exact test). A large proportion of these patients also received chemotherapy: 18 patients (66.6 %) in group 1 and 8 patients (72.7 %) in group 2 (p = 1.0, fisher's exact test).

When evaluating the MRI datasets for group 1, diffusion sequences were present in 26 out of 27 patients. In group 2, they were present in nine of the 11 patients.

**Fig. 4:**
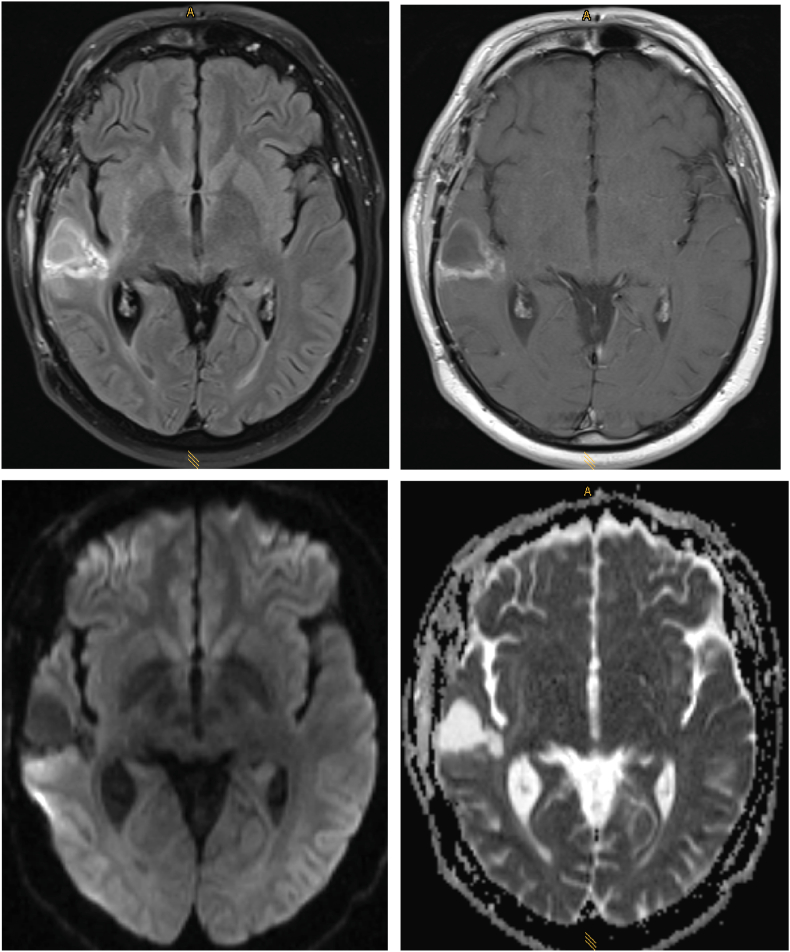
Imaging findings of a histologically confirmed foreign body granuloma, mimicking glioma recurrence Fluid attenuated inversion recovery and T1weighted axial images showing a right-hand side temporal lesion with T2 prolongation and rim-enhancement in follow up imaging. The diffusion weigthed image (b1000) and the corresponding apparent diffusion coefficent (ADC) map show restricted diffusion, represented by increased b1000 signal and decreased ADC within the contrast enhancing portion of the lesion, suggestive of an inflammatory process.

**Fig. 5:**
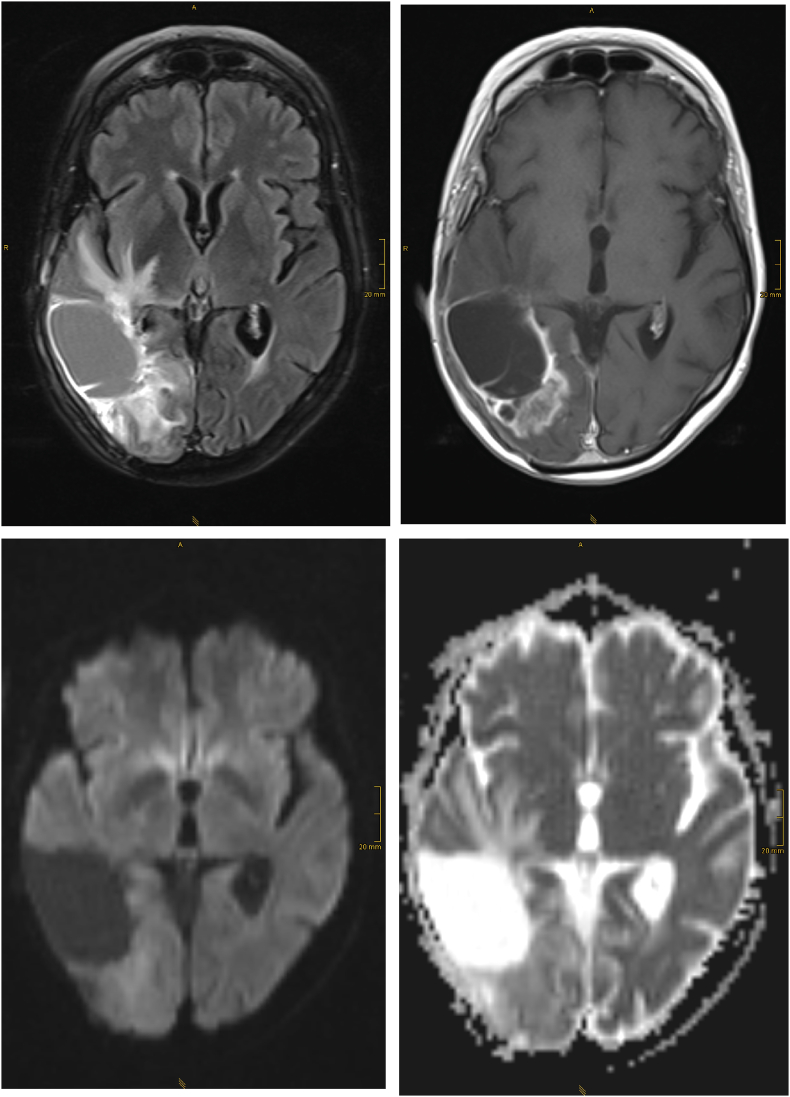
Imaging findings of histologically confirmed recurrent high-grade glioma. Fluid attenuated inversion recovery and T1weighted axial post contrast images showing a right-hand side temporal lesion with T2 prolongation and rim-enhancement in follow up imaging. The diffusion weighted image (b1000) and the corresponding apparent diffusion coefficent (ADC) map show slightly enhanced diffusion, represented by an increased ADC with T2shine through in the contrast enhancing portion of the lesion, suggestive for tumor recurrence.

Restrictions in diffusion were observed, characterized by a decrease in ADC values of varying severity. The mean ADC value was 812.603 × 10^−6^ mm^2^/s (SD 142.2) in group 1 and 673.87 **x** 10^.−6^ mm^2^/s (SD 86.8) in group 2. Difference did not reach statistical significance (p = 0.52 and p = 0.19, Mann-Whitney-U-test). Fig. 4, Fig. 5 show the MRI of a representative case of true recurrence and a case without evidence of tumor tissue, respectively.

Of the reoperated patients who were originally treated with Tabotamp® (84 %), no tumor could be detected in one third after the relapse operation. Using the RANO criteria, all 10 patients in whom no tumor could be detected histologically were found to have progressive disease.

## 4 Discussion

Foreign body granulomas (FBG) following neurosurgical procedures represent a rare but clinically and diagnostically significant differential diagnosis compared to tumor recurrence. Especially in patients with gliomas, who are typically monitored closely during follow-up, FBG can easily be misinterpreted as progression or recurrence (Anderson et al., 2008; Winter et al., 2021). The prevalence is unclear and probably underestimated, as FBG are often asymptomatic and therefore often only discovered as incidental findings (Winter et al., 2021). At the same time, FBG are not limited to glioma surgery, but have also been documented after other intracranial procedures, such as vascular surgery (Dinesh et al., 2008). Pathophysiologically, there is consensus that exogenous material such as cotton fibers, suture material, or bone wax can induce granulomatous inflammatory reactions (Nakayama et al., 1994; Forst et al., 2016; Al-Afif et al., 2018). In our view, it is important to consider FBG as a differential diagnosis at an early stage, in order to avoid unnecessary procedures that can pose risks and complications for patients. However, achieving the right balance between a wait-and-see approach and further treatment will be challenging.

Conceptually, the challenge is that the radiological features of FBG are by no means clear-cut, and the literature even describes contradictory findings. Some studies have reported diffusion restriction in DWI (Winter et al., 2021), an imaging feature that is usually associated with high cell density and thus tumor activity. Other studies, however, have predominantly found no diffusion restriction (Lin et al., 2021). It is likely that this apparent paradox can be explained by heterogeneity in material, quantity, and time interval at the time of imaging: the cellular component of an FBG can vary depending on the stage (early inflammatory vs. later fibrotic), which is reflected differently in DWI.

In contrast to DWI, perfusion-based methods provide more stable resolution. FBG rarely show a relevant increase in rCBV (Jang et al., 2010) and MR spectroscopy is typically only slightly increased in favor of choline (Jang et al., 2010)). These findings are rather atypical for malignant progression. The radiological findings are therefore benign overall — but not necessarily sufficient to rule out malignancy, as “shine through” effects, for example due to contrast agent residues in cavities or residual tumors in the immediate vicinity, can make interpretation difficult. Diagnostic uncertainty is also exacerbated by nuclear medicine procedures: [^18^F]fluorocholine PET/CT can be false positive in FBG and thus mistakenly mimic progression (Rudolphi-Solero et al., 2023).

This has clear implications for clinical practice: FBG must be systematically considered as a relevant differential diagnosis in new lesions in the postoperative course of glioma patients. This is particularly true if the findings do not translate into a coherent overall picture of progression. In particular, combinations of: (i) no increase in rCBV (relative cerebral blood volume), (ii) only minimal increase in choline in MR spectroscopy, (iii) moderate or no diffusion restriction, and (iv) absence of clinical symptoms should raise the threshold for premature assessment of recurrence.

In cases of uncertainty, a wait-and-see approach with close monitoring is justifiable and often advisable before irreversible therapeutic steps are taken. This is because the consequences of a misdiagnosis are considerable: escalation to chemotherapy or re-irradiation, or even reoperation, carries morbidity-related risks and must be weighed against the possibility of a benign process. Multidisciplinary discussion in tumor boards/neuroradiology conferences is therefore essential.

The following appear to be particularly relevant for the future: (i) prospective systematic recording of foreign materials used intraoperatively, (ii) standardized imaging documentation from defined points in time postoperatively, and (iii) a methodical correlation of imaging phenotypes with material type and time course. In the medium term, this could lead to the development of a diagnostic algorithm that validates the decision tree for biopsy or conservative follow-up.

Overall, this analysis underscores that although the existence of FBG after glioma surgery is rare, it is real and highly relevant. Consciously integrating this into diagnostic thinking can prevent misdiagnoses, improve treatment decisions, and potentially avoid unnecessary interventions.

## 5 Conclusion

FBG is a radiological heterogeneous phenomenon and must be considered as significant, although rare differential diagnosis after tumor resection. Conventional images oftentimes are not helpful to distinguish between tumor recurrences and FBG. However, additional techniques, such as DWI and PWI can provide additional clues in this regard, and the presence of diffusion restriction within a contrast enhancing lesion after surgery should be considered a potential FBG.

## Declaration of competing interest

The authors declare that they have no known competing financial interests or personal relationships that could have appeared to influence the work reported in this paper.
