# Supplementary material for: Not all enhancing lesions are tumor recurrence: Foreign body granulomas after glioma resection
Source: Brain Spine. 2026 Jun 23;6:106140. doi: 10.1016/j.bas.2026.106140 (PMC13316632; doi:10.1016/j.bas.2026.106140)
Supplement: Multimedia component 1 [file mmc1.docx]

**Table S1.** Clinical Characteristics, Progression-Free Survival, and Overall Survival (months) of Patients with Histologically Confirmed Tumor at Reoperation

| **Case** | **Initial Tumor grading** | **PFS 1** | **Recurrent Tumor grading** | **PFS 2** | **Vital status at last Follow-Up** | **OS** |
| --- | --- | --- | --- | --- | --- | --- |
| 1 | 4 | 4.3 | 4 |  | Dead | 14.3 |
| 2 | 4 | 8.1 | 4 |  | Dead | 16.1 |
| 3 | 4 | 8.1 | 4 |  | Dead | 16 |
| 4 | 4 | 8.9 | 4 |  | Dead | 27.8 |
| 5 | 4 | 4.7 | 4 |  | Dead | 8.9 |
| 6 | 4 | 9.1 | 4 |  | Dead | 23.2 |
| 7 | 4 | 13.6 | 4 | 21.2 | Dead | 32 |
| 8 | 4 | 3.3 | 4 | 46.8 | Alive | 85* |
| 9 | 4 | 4.4 | 4 | 15.7 | Dead | 22.7 |
| 10 | 4 | 13.4 | 4 | 19.7 | Dead | 24.5 |
| 11 | 4 | 1.9 | 4 |  | Dead | 8.9 |
| 12 | 4 | 19.6 | 4 |  | Dead | 31.7 |
| 13 | 4 | 23.4 | 4 | 24.2 | Dead | 27.3 |
| 14 | 4 | 4.3 | 4 |  | Dead | 11.3 |
| 15 | 4 | 18 | 4 |  | Dead | 38.9 |
| 16 | 4 | 12.1 | 4 | 15.3 | Dead | 18.9 |
| 17 | 4 | 17.4 | 4 |  | Dead | 22 |
| 18 | 3 | 96.4 | 4 | 113.9 | Dead | 156.5 |
| 19 | 3 | 11 | 4 | 13.3 | Dead | 15.9 |
| 20 | 3 | 51.6 | 3 | 71.6 | Alive | 192* |
| 21 | 2 | 20.6 | 3 | 35.1 | Dead | 45.1 |
| 22 | 2 | 24.7 | 2 |  | Alive | 107* |
| 23 | 2 | 16,3 | 4 |  | Dead | 18.5 |
| 24 | 2 | 10 | 2 |  | Alive | 113* |
| 25 | 2 | 9,1 | 2 |  | Alive | 116* |
| 26 | 2 | 25,2 | 2 |  | Alive | 133* |
| 27 | 2 | 44,6 | 4 |  | Dead | 49.6 |

**Table S2.** Clinical Characteristics, Progression-Free Survival, and Overall Survival (months) of Patients without Histological Evidence of Tumor at Reoperation

| **Case** | **Initial Tumor grading** | **PFS 1** | **Recurrent Tumor grading** | **PFS 2** | **Vital Status at last Follow-Up** | **OS** |
| --- | --- | --- | --- | --- | --- | --- |
| 1 | 4 | 5.8 | 4 | 25.8 | Dead | 33.6 |
| 2 | 4 | 1.5 | 4 | 7.1 | Dead | 10 |
| 3 | 4 | 45.1 | 4 |  | Dead | 55.9 |
| 4 | 4 | 5.7 | 4 |  | Dead | 13.5 |
| 5 | 4 | 49.2 | 4 | 56 | Dead | 61.5 |
| 6 | 4 | 12.6 | 4 |  | Dead | 16.9 |
| 7 | 4 | 4.6 | 4 | 9.8 | Lost to follow up | / |
| 8 | 3 | 92.3 | 4 | 188.4 | Alive | 204* |
| 9 | 2 | 0 | 2 |  | Alive | 93* |
| 10 | 2 | 9.6 | 2 | 41.7 | Alive | 117* |
| 11 | 2 | 65.6 | 2 | 122.9 | Alive | 175* |
